# Pulmonary artery enlargement in schistosomiasis associated pulmonary arterial hypertension

**DOI:** 10.1186/s12890-015-0115-y

**Published:** 2015-10-12

**Authors:** Susana Hoette, Claudia Figueiredo, Bruno Dias, Jose Leonidas Alves-Jr, Francisca Gavilanes, Luis Felipe Prada, Dany Jasinowodolinski, Luciana Tamie Kato Morinaga, Carlos Jardim, Caio Julio Cesar Fernandes, Rogério Souza

**Affiliations:** Pulmonary Department – Heart Institute, University of São Paulo Medical School, Av. Dr. Eneas de Carvalho Aguiar, 44, Sao Paulo, 05403-000 Brazil

**Keywords:** Schistosomiasis, Idiopathic pulmonary arterial hypertension, Pulmonary artery, Hemodynamics, Computed tomography

## Abstract

**Background:**

Schistosomiasis associated pulmonary arterial hypertension (Sch-PAH) might represent the most prevalent form of PAH worldwide. In Sch-PAH, the presence of aneurismal dilation of the pulmonary artery has been described, although it is still a matter of debate whether on average the pulmonary artery is more enlarged in Sc-PAH than IPAH.

**Methods:**

We retrospectively evaluated patients with IPAH (*n* = 25) and Sch-PAH (*n* = 22) who underwent computed tomography pulmonary angiogram and right heart catheterization.

**Results:**

Sch-PAH patients were older and presented less severe hemodynamic profiles. Main pulmonary artery diameter (MPAD) was greater in Sch-PAH than IPAH (4.5 ± 1.8 vs 3.7 ± 1.1 cm, *p* = 0.018). For the same level of mean pulmonary artery pressure, the MPAD in Sch-PAH was 0.89 cm larger than in IPAH (Covariance model *p* = 0.02).

**Conclusion:**

This study demonstrated that pulmonary artery enlargement is more pronounced in Sch-PAH than IPAH, independently of mean pulmonary artery pressure level, suggesting that this is more likely a feature of Sch-PAH.

## Background

Schistosomiasis associated pulmonary arterial hypertension (Sch-PAH) has gained attention in recent years because it might represent the most prevalent form of PAH worldwide [1, 2]. In Brazil, it represents approximately 20 % of all PAH patients [3]. Although Sch-PAH has a similar clinical and pathological presentation as idiopathic PAH (IPAH), the clinical course seems to be more benign, with a three-year mortality of 15 % [4, 5]. Even though an increased amount of data regarding Sch-PAH has been published in recent years, little is known about the radiological features of this particular form of PAH.

In patients with pulmonary hypertension, the main pulmonary artery diameter (MPAD) is significantly increased when compared to controls. Badagliacca et al. [6] demonstrated that 76.6 % of a cohort of PH patients had dilation of the pulmonary artery (PA). Patients with congenital heart disease had a larger PA diameter size compared to other PH groups [7]. In patients with Sch-PAH, the presence of aneurismal dilation of the PA has been described, although it is still matter of debate whether or not this is a feature of Sch-PAH or a casual finding [8]. The aim of this study was to compare the computed tomography pulmonary angiogram (CTPA) features of Sch-PAH patients to those of patients with IPAH.

## Methods

We retrospectively evaluated charts from IPAH and Sch-PAH patients who underwent CTPA and right heart catheterization (RHC) in our institution, as part of their baseline evaluation, without the use of any targeted therapy. The diagnosis of IPAH was established when other causes and associated diseases were excluded as described in previous studies [9]. Patients were classified as having Sch-PAH when the presence of PAH was associated with liver ultrasonographic findings highly suggestive of mansonic schistosomiasis (left lobe enlargement and/or periportal fibrosis) and at least one of the following features: (1) exposure to endemic region for schistosomiasis; (2) previous treatment of schistosomiasis; and (3) presence of *Schistossoma mansoni* eggs in stool examination or rectal biopsy [4].

CTPAs were reanalyzed by a chest radiologist blinded to the final diagnosis and hemodynamic profiles. Diameter measurements were obtained from the pulmonary trunk, left and right PAs. MPAD was defined as the largest diameter of the pulmonary trunk obtained from its origin to 3 cm above its bifurcation. The right pulmonary artery diameter (RPAD) was measured in its horizontal portion, in a midline plane, posterior to the ascending aorta and superior vena cava. The left pulmonary artery diameter (LPAD) was measured in its inflexion, where it crosses over the left upper lobe bronchus. The protocol was approved by the Institutional Review Board of the University of Sao Paulo Medical School Hospital (HCFMUSP - protocol number 10303) without requiring informed consent due to the retrospective nature of the study.

## Statistical analysis

Continuous variables were expressed as mean ± SD and compared using unpaired t-tests. Categorical variables were expressed as proportions and compared using Fisher’s exact test. Normality of the distribution in both samples was tested using Kolmogorov-Smirnov test. Pearson correlation test was used to evaluate the association of hemodynamic variables and main pulmonary artery diameter. Covariance analysis was used for the comparison of the MPAD, correcting for the level of mean pulmonary artery pressure (mPAP). A *p*-value of 0.05 was considered to be significant.

## Results

The study sample consisted of 47 patients: 25 with IPAH and 22 with Sch-PAH. Sch-PAH patients were older (48 ± 13 vs 40 ± 13 years-old in IPAH, *p* = 0.02), as previously described [5], but gender distribution and functional status were similar between the two groups. Moreover, Sch-PAH patients presented a less severe hemodynamic profile than the IPAH patients (Table 1).

**Table 1:** Demographics, hemodynamic and CTPA data

	IPAH	Sch-PAH	*p*
Gender			
Male	4 (16 %)	4 (18 %)	ns
Female	21 (84 %)	18 (81 %)	
Age	40 ± 13	48 ± 13	0.019
Functional class			
I/II	7 (28 %)	7 (32 %)	ns
III/IV	18 (72 %)	15 (68 %)	
Hemodynamics			
mPAP (mmHg)	63 ± 23	56 ± 23	ns
RAP (mmHg)	15 ± 11	9 ± 4	0.018
PAOP (mmHg)	12 ± 6	15 ± 7	ns
PVR (IU)	15.4 ± 5.1	10.2 ± 6.1	0.009
CO (mL/min)	4.9 ± 1.8	3.8 ± 1.2	0.03
CTPA measurements			
MPAD, cm	3.7 ± 1.1	4.5 ± 1.8	0.018
RPAD, cm	2.58 ± 0.47	3.16 ± 0.91	0.009
LPAD, cm	2.5 ± 0.4	2.7 ± 0.6	ns

*IPAH* idiopathic pulmonary arterial hypertension, *Sch-PAH* schistosomiasis associated pulmonary hypertension, *NYHA FC* New York Heart Association functional class, *mPAP* mean pulmonary arterial pressure, *RAP* right atrial pressure, *PAOP* pulmonary artery occlusion pressure, *PVR* pulmonary vascular resistance, *CO* cardiac output, *MPAD* main pulmonary artery diameter, *RPAD* right pulmonary artery diameter, *LPAD* left pulmonary artery diameter, *ns* non significant

**Fig. 1:**
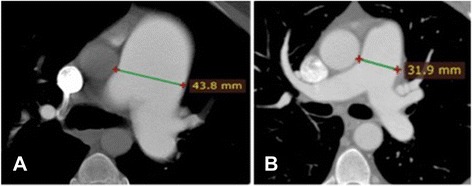
CTPA showing the measurement of the MPAD in a Sch-PAH patient (**a**) and in a IPAH patient (**b**). Sch-PAH patient has a larger MPAD (43.8 mm) despite of having lower mPAP values (patient **a** - mPAP of 57 mmHg; patient **b** - mPAP of 75 mmHg)

**Fig. 2:**
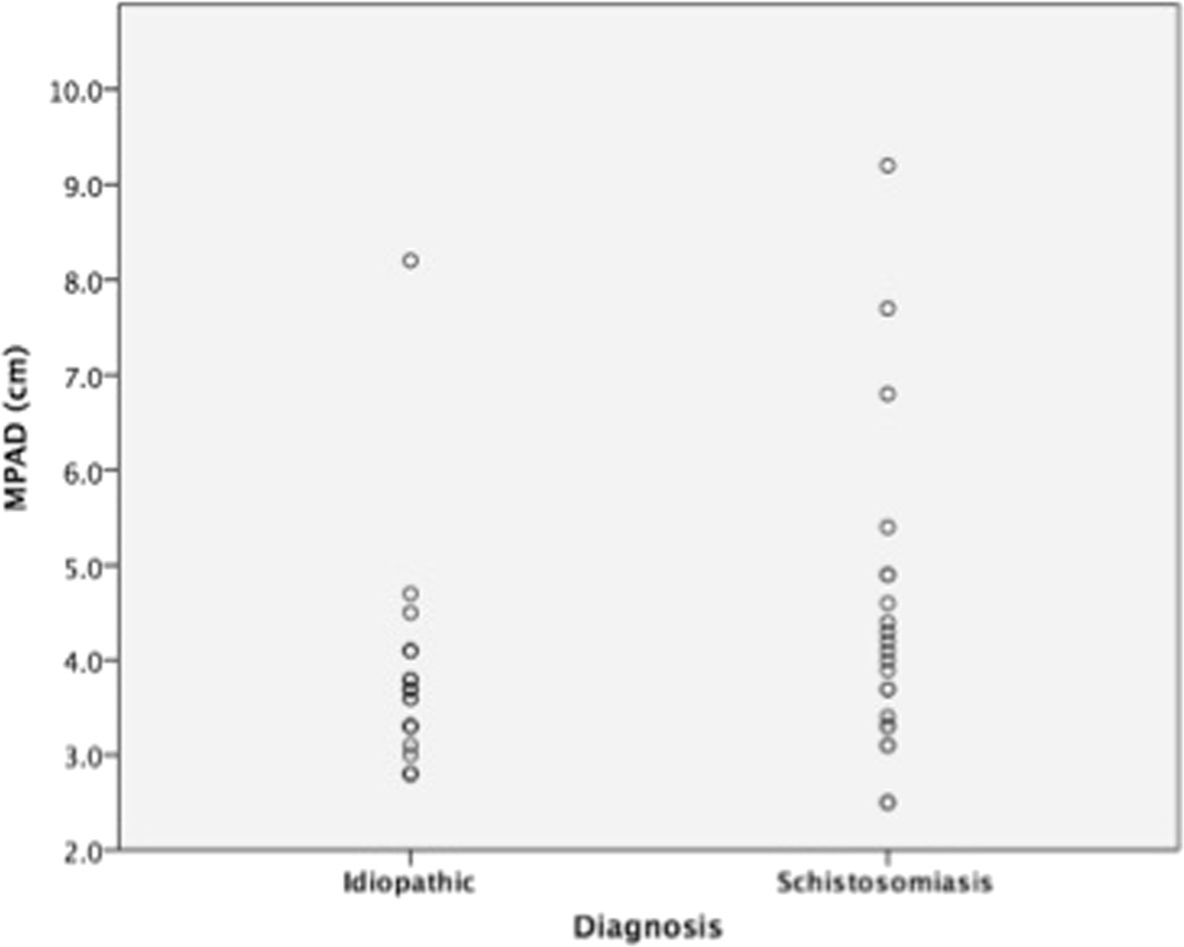
Distribution of MPAD according to diagnosis

**Fig. 3:**
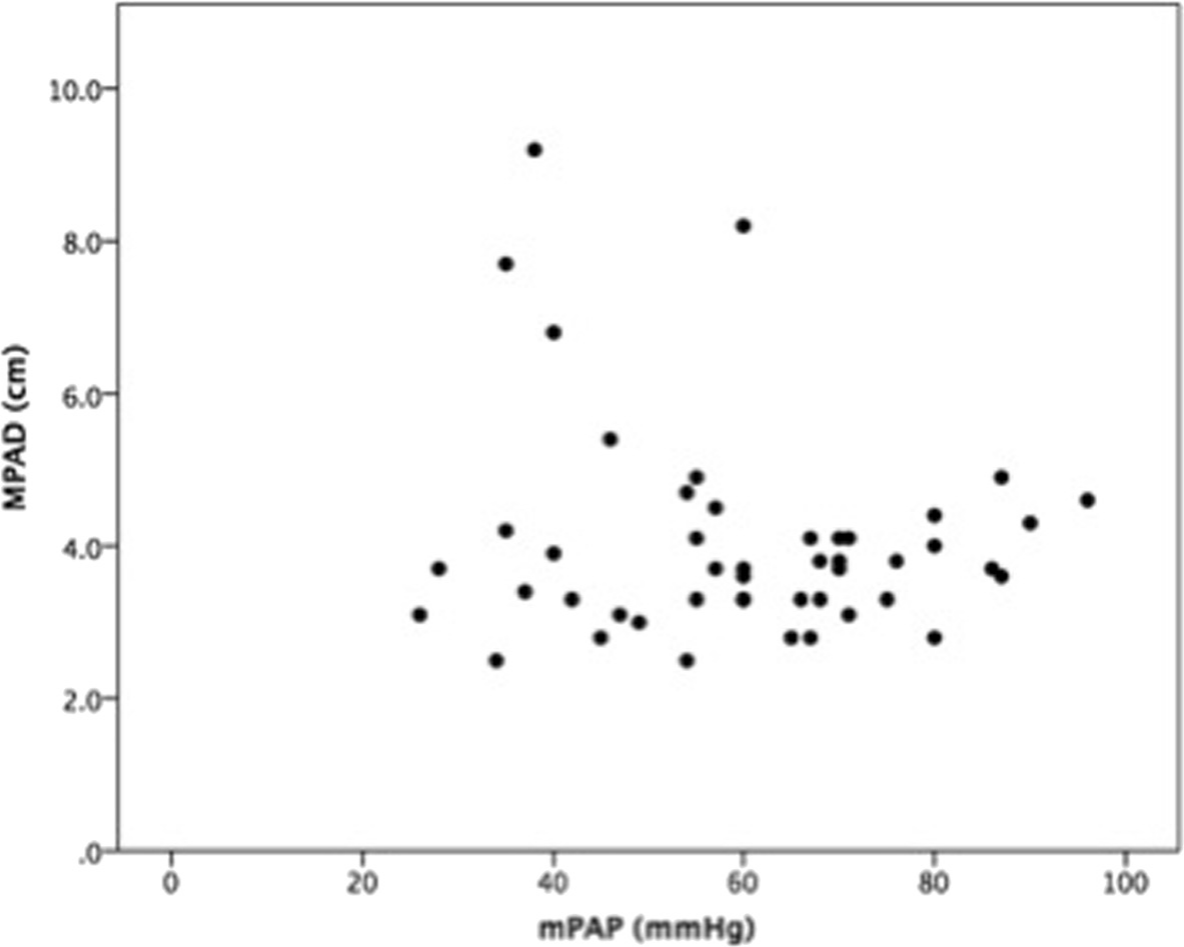
Correlation between MPAD and mPAP

MPAD was significantly greater in Sch-PAH than IPAH patients (4.5 ± 1.8 vs 3.7 ± 1.1 cm, *p* = 0.018) (Fig. 1). The distribution of MPAD according to diagnosis is demonstrated in Fig. 2. RPAD was also significantly larger in patients with Sch-PAH (3.2 ± 0.9 vs 2.6 ± 0.5 cm, *p* = 0.009). LPAD was larger in the Sch-PAH group although not significantly (2.7 ± 0.6 vs 2.5 ± 0.4 cm, *p* = 0.098). There was no significant correlation between MPAD and mPAP (Fig. 3) or any other hemodynamic variable.

To avoid bias that might occur due to a different hemodynamic profile, we performed an analysis of covariance, correcting the comparison of the MPAD for the level of mPAP. For the same level of mPAP, the MPAD in Sch-PAH is 0.89 cm larger than in IPAH (Covariance model *p* = 0.02).

## Discussion

This study demonstrated that PA enlargement is more pronounced in Sch-PAH than in IPAH patients, independently of mPAP level, suggesting that this is a characteristic feature of Sch-PAH.

PA involvement in schistosomiasis is not unusual. Andrade et al. [10] discovered PA abnormalities in 18.9 % of 232 necropsies of patients with severe hepatosplenic schistosomiasis. Particularly in the setting of pulmonary hypertension, the histological alterations found in Sch-PAH patients are indistinguishable from those found in IPAH patients, with the exception of the occasional finding of granulomas, which are not found in IPAH [5]. Given its histological similarities with IPAH, and also its similar clinical characteristics and response to targeted therapies [5, 11], Sch-PAH, previously classified in group IV (embolic diseases), was changed to group I (PAH) [12].

In the present study, we demonstrated that patients with Sch-PAH have a more pronounced dilation of the PA than patients with IPAH. The reason for this finding is not known. A study by Boerrigter et al. [13] compared cardiac magnetic resonance data of patients with PH before and after treatment, with a mean follow-up time of 942 days. They observed a significant increase in cardiac output and a significant decrease in pulmonary vascular resistance after treatment. Despite this hemodynamic improvement, there was a significant increase in MPAD. Therefore, no relationship between hemodynamic improvement and MPAD was observed. In fact, MPAD increased with time, suggesting that this enlargement could be more related to the duration of the disease than to the severity of the hemodynamic profile in PAH patients. As suggested by the authors, dilation of the PA may be related to intrinsic properties of the PA wall. Accordingly, our study demonstrated that even after correcting for the difference of pressure, Sch-PAH patients had more pronounced PA dilations.

The hypothesis that dilation of the main pulmonary artery represents an adaptive response is supported by two case reports: one describes two patients with IPAH with an unusually long survival with markedly dilated PAs (RPA of 4.6 and 8.3 cm) [14] and the second [15] describes three cases of long-lasting PAH that had aneurismal dilation of the PA. The marked dilation of the PAs could represent an adaptive response aiming to increase vascular capacitance in order to optimize hemodynamics and reduce right ventricular (RV) overload, which may be an attempt to preserve RV function.

Concomitant finding of PA enlargement and better long-term outcome is commonly seen in patients with PAH associated to congenital heart disease [6, 15]. Interestingly, Fernandes et al. [5] demonstrated that Sch-PAH patients had more benign clinical courses than IPAH patients, with improved survival. Our results evidenced greater MPAD in these patients; therefore, longer survival could be one explanation for the pronounced dilation of the PAs in this subgroup of PAH. Nevertheless, the presence of a specific adaptive vascular change or the influence of other pathophysiological mechanisms leading to pulmonary artery dilation and also to better long-term survival cannot be ruled out.

Inflammation and endothelial dysfunction have been demonstrated to be involved in the pathogenesis of PAH. Mononuclear inflammatory infiltrates, lymphocytes and machophages are present surrounding the vascular site of plexiform growth. Increased levels of proinflammatory cytokines interleukin (IL-1, IL-6) and exaggerated production of chemokines have been reported in patients with IPAH [16, 17]. In schistosomiasis, inflammation is also an important factor. The acute phase of the disease is characterized by a type 1 T cell response (Th1) with the predominance of IL-1, INF-γ, TGF-β, TNF-α. In the chronic phase, schistosome eggs provide a chronic antigenic stimulus for the immune response, and there is a switch to a type 2 T cell response (Th2). During this phase, IL-4, IL-5, IL-10 and IL-13 secretion predominates [18]. Granuloma macrophages are also responsible for maintaining the chronic inflammatory response. Studies comparing the inflammatory response in patients with IPAH and Sch-PAH are still lacking. Lapa et al. [19], however, demonstrated that patients with IPAH had increased levels of E-selectin when compared to patients with Sch-PAH. This finding suggests that the endothelium impairment in Sch-PAH might involve different pathways than in IPAH. A difference in inflammatory response and endothelial dysfunction between these two forms of PAH may lead to different changes in the PA wall that might explain the difference found in the patterns of PA dilation.

This study has limitations that need to be acknowledged. This was a single center study in which baseline and hemodynamic data were collected retrospectively limiting the availability of some important variables like duration of the disease; the demonstration of different duration of the disease development would further strength the hypothesis that this could be the main reason for the differences found in our study. In fact, the finding of a significant number of patients with important enlargements in PA might already suggest chronicity of these changes. Hemodynamic study was not performed on the same day as CTPA; nevertheless, no therapeutic interventions or clinical deterioration existed in the interval between the two exams. In addition, patients with Sch-PAH were significantly older than patients with IPAH; however, observed differences remained significant even after correcting for age. It is important to highlight that, as in other forms of pulmonary hypertension, the definition of Sch-PAH is based on empirical criteria; nevertheless, these criteria were used in order to raise the chance of association between PAH and schistosomiasis.

## Conclusion

In conclusion, significant PA dilatations are more likely a feature of Sch-PAH that should raise the diagnostic suspicion in PAH patients coming from endemic regions for schistosomiasis.
